# Role of plants in the transmission of *Asaia* sp., which potentially inhibit the *Plasmodium* sporogenic cycle in *Anopheles* mosquitoes

**DOI:** 10.1038/s41598-020-64163-5

**Published:** 2020-04-28

**Authors:** Hubert Bassene, El Hadji Amadou Niang, Florence Fenollar, Souleymane Doucoure, Ousmane Faye, Didier Raoult, Cheikh Sokhna, Oleg Mediannikov

**Affiliations:** 1 VITROME, Campus International UCAD-IRD, Dakar, Sénégal; 2 MEФI, IRD, Aix Marseille Univ, AP-HM, Marseille, France; 3 IHU-Méditerranée Infection, Marseille, France; 4 Laboratoire d’Ecologie Vectorielle et Parasitaire, Faculté des Sciences et Techniques, Université Cheikh Anta Diop (UCAD), de Dakar, Sénégal; 5 VITROME, Aix Marseille Univ, IRD, AP-HM, SSA, Marseille, France

**Keywords:** Microbial ecology, Bacteriology, Entomology

## Abstract

Biological control against malaria and its transmission is currently a considerable challenge. Plant-associated bacteria of the genus *Asaia* are frequently found in nectarivorous arthropods, they thought to have a natural indirect action on the development of plasmodium in mosquitoes. However, virtually nothing is known about its natural cycle. Here, we show the role of nectar-producing plants in the hosting and dissemination of *Asaia*. We isolated *Asaia* strains from wild mosquitoes and flowers in Senegal and demonstrated the transmission of the bacteria from infected mosquitoes to sterile flowers and then to 26.6% of noninfected mosquitoes through nectar feeding. Thus, nectar-producing plants may naturally acquire *Asaia* and then colonize *Anopheles* mosquitoes through food-borne contamination. Finally, *Asaia* may play an indirect role in the reduction in the vectorial capacity of *Anopheles* mosquitoes in a natural environment (due to *Plasmodium*-antagonistic capacities of *Asaia*) and be used in the development of tools for *Asaia*-based paratransgenetic malaria control.

## Introduction

In the context of malaria pre-elimination, the emergence of resistance in both vectors and parasites threatens the progress made in recent decades. The search for alternative methods is becoming a priority for the WHO and the scientific community. The vector environment has very often been neglected during the implementation of control strategies.

The present study focuses on the role of nectar-producing plants in reducing the vectorial capacity of malaria-transmitting mosquitoes. We found that flower nectar is home to viable strains of *Asaia*, known for their natural antagonism to *Plasmodium falciparum*, and we isolated *Asaia* strains from *Ipomoea pes-caprae*. Moreover, we found that bacterial exchanges between plants and mosquitoes are common in the natural environment and thus discovered the natural *Asaia*/*Anopheles*/flower nectar cycle. Through these exchanges, plants, as the habitat for *Plasmodium*-suppressing *Asaia* bacteria, may contribute to the reduction in the vectorial capacity of *Anopheles* (which has already been empirically reported). It was recently reported that an *Asaia*-based paratransgenetic malaria control strategy may also rely on the *Asaia* natural cycle as a delivery tool.

Particular attention should be paid to the environment in which mosquitoes are founding the development of malaria control strategies.

Malaria continues to be one of the most common causes of death on earth. In addition, its transmission is only partially controlled due to the risk of the emergence of insecticide-resistant mosquitoes. This information justifies the search for alternative and nonpolluting solutions to control mosquito-borne infections, especially since, with the risk of global warming, the area affected by malaria may increase. In this context, the search for biological control agents against malaria and its transmission is a considerable challenge. The simultaneous presence of *Asaia* and *Plasmodium falciparum* has not yet been reported in mosquitoes suggesting antagonism^1,2^. Thus, identifying the source of *Asaia* contamination in *Anopheles* may help to determine how to apply biological control to limit *Anopheles* infestation by *P. falciparum*. Recently, a paratransgenetic strategy was proposed based on *Asaia* bacteria that conditionally expressed an anti-plasmodial protein only when a blood meal was present^3^.

Nectarivory is extremely common in insects. Nectar is a sugar-rich liquid specially produced by plant glands (nectaries) to attract pollinating animals. The majority of nectarivorous insect species belong to the families *Diptera*, *Coleoptera*, *Lepidoptera*, *Hymenoptera* and *Hemiptera*. Nectar consumption has been established for many lower dipteran taxa, including not only the *Tipulidae* (crane flies), *Cecidomyiidae* (gall midges), *Sciaridae* (root gnats) and *Mycetophilidae* but also blood-feeding species, such as *Ceratopogonidae* (biting midges) and *Culicidae* (mosquitoes)^4^. The *Diptera* possess an elongated tube, which has characteristic morphological and functional adaptations to feed on either floral nectar (e.g., *Bombyliidae*, *Syrphidae*, and *Nemestrinidae*), vertebrate blood (e.g., *Tabanidae* and *Glossinidae*) or both (*Culicidae*, *Simuliidae*, and *Ceratopogonidae*)^5^.

Male and female mosquitoes require sugar to obtain the energy they need to survive in their natural environment. Their sugar requirements are generally fulfilled from flower nectar near larval breeding sites or around adult resting sites. Plant nectar is a nutritional supplement that females often use between blood meals and when hosts are absent or rare. It is also used as an energy source and thus allows both male and female mosquitoes to survive in their environment^6^. Thus, plants play a very important role in mosquito survival in their natural environment, providing them with an easily accessible meal, which increases their life span and reduces contact with humans^6^. Plants also provide male mosquitoes with enough energy to successfully fertilize females, thus ensuring species perennity^7^. Mosquitoes are attracted by the odors emitted by plants and use those odors to locate plants that can provide the sugar and/or chemicals they need to survive in their natural environment^6^.

Several studies have discussed the importance of arthropod vector microbiota for the prevention of certain vector-borne diseases^8–11^. The composition of these microbiota is not only influenced by the bacterial flora in breeding sites but also by the sources of adult mosquitoes’ diets^12,13^. Some bacteria from larval breeding sites or plants can be absorbed by mosquitoes when ingesting food^12,14^.

Although highly suspected, very little data are available regarding the potential of flower nectar to facilitate the food-borne transmission of microorganisms between blood-feeding dipterans. Indeed, until a flower opens, the nectar inside of it remains sterile; however, once a flower opens, the nectar can be contaminated and serve as a source of contamination for all the insects that feed on it. We therefore hypothesized that plants are the key link between mosquitoes and the mosquito-borne transmission of *Asaia* spp. *Asaia* bacteria were isolated for the first time from the flowers of the tropical plants *Bauhinia purpurea* and *Plumbago auriculata*^15–18^. In such cases, plants may indirectly play a negative role in the transmission of vector-borne diseases, such as malaria, by being reservoirs and fomites of *Plasmodium*-antagonistic bacteria such as *Asaia*^2^.

Indeed, the absorption of nectar from plants has already been reported to have negative effects on the transmission of certain diseases^14^. According to Hien *et al*., 2016, three mutually nonexclusive mechanisms could explain the effects of plant sugar on the ability of a vector to transmit *P. falciparum*^19^. First, the ingestion of secondary metabolites, such as alkaloids, terpenes and glycosides, has a negative effect on *Plasmodium* development. Indeed, these chemicals, present in all plant tissues, including flower nectar and fleshy fruits, are toxic to pests and reduce their life span and reproductive capacity and, therefore, play a key role in plants’ defense system against pests^20,21^. Second, feeding on poorly energetic plants could induce changes in the metabolism of mosquitoes and result in an energy deficiency that would limit the development of the parasite in the vector^22^. Third, plants can indirectly influence the development of parasites in mosquitoes through their effects on the immune system or gut microbiota. A deficit of energy would cause a dysfunction of the immune system and a reduction in the defenses against the parasite^23^. However, until now, the role of plants in the transmission of bacteria such as *Asaia* spp. and/or other microorganisms that have an antagonistic effect on *Plasmodium* sp. has not been described.

This study therefore aims to identify the role of plant nectar in the epidemiological cycle *of Asaia* bacteria and to test the hypothesis that plants act as a reservoir from which wild mosquitoes are constantly infected.

## Results

### Antibiogram and selective medium preparation

The resistance status of the previously isolated *A. aff. bogorensis* GD01 strain^2^ to different antibiotics was assessed (Table 1). The data obtained were then used to prepare an antibiotic-containing Columbia-based selective solid medium for the isolation of *Asaia* strains from naturally infected flowers and to obtain an *Asaia*-free mosquito line from a laboratory-reared colony of *An. coluzzii*.

**Table 1:** Sensitivity *of A. aff. Bogorensis* GD01 to antibiotics.

Family	Molecule	Disc load	Signs	Critical concentration (mg/L)		Critical diameter (mm)		Total diameter read (mm)	Status
				S	R	S	R		
Penicillins	Amoxicillin	25 µg	AX 25	≤2	>8	≥23	<16	7	Resistant
	Amoxicillin/clavulanic acid	20 µg/10 µg	AMC 30	≤2/2	>8/2	≥23	<16	10	Resistant
	Piperacillin/tazobactam	75 µg/10 µg	TPZ 85	≤4	>16	≥22	<18	22	**Sensitive**
Carbapenems	Doripenem	10 µg	DOR 10	≤1	>4	≥24	<19	34	**Sensitive**
	Imipenem	10 µg	IPM 10	≤2	>8	≥24	<17	35	**Sensitive**
Monobactams	Aztreonam	30 µg	ATM 30	≤4	>8	≥23	<21	8	Resistant
Cephalosporins	Ceftriaxone	30 µg	CRO 30	≤1	>2	≥26	<23	14	Resistant
	Ceftazidime	30 µg	CAZ 30	≤4	>8	≥21	<19	10	Resistant
	Cefpirome	30 µg	CPO 30	≤4	>8	≥21	<19	12	Resistant
Aminosides	Gentamicin	15 µg/10 IU	CN 15	≤2	>4	≥18	<16	16	Resistant
Tetracyclines	Doxycycline	30 IU	DO 30	≤4	>8	≥19	<17	27	**Sensitive**
Macrolides	Erythromycin	15 IU	E 15	≤1	>4	≥22	<17	17	Resistant
Sulfamides-Trimethoprim	Trimethoprim/sulfamethoxazole	1,25 µg/23.75 µg	SXT 25	≤2/38	>8/152	≥16	<10	0	Resistant
Fluoroquinolones	Ciprofloxacin	5 µg	CIP 5	≤0,5	>1	≥25	<22	18	Resistant
Oxazolidinones	Linezolid	30 µg	LNZ 30	≤2	>4	≥28	<24	23	Resistant
Other families	Metronidazole	4 µg	MET 4	≤4	>4		<21	12	Resistant
	Rifampicin	30 µg	RA 30	≤4	>16	≥19	<14	17	Resistant

Abbreviations: S = Sensitive, R = Resistant.

The families to which the antibiotics that have been used belong are listed in the column Family. The different antibiotics used are listed in the Molecule column. The disc loads are provided in the Disc load column. The antibiotic acronyms are listed in the Signs column. The minimum (**S**) and maximum (**R**) average concentrations obtained for each antibiotic are listed in the Critical concentration column and are expressed in milligrams per liter (mg/L). The diameters beyond which the strains are considered sensitive (**S**) or resistant (**R**) are provided in the Critical diameters column; these diameters are measured in millimeters (mm). The diameters read after incubation of the bacterial cultures are given in the Total diameter read column, and the values are measured in millimeters (mm). Finally, the interpretation of the results is shown in the Status column.

In summary, *A. aff. bogorensis* GD01 was resistant to most families of the antibiotics tested (Table 1), except piperacillin-tazobactam, doxycycline, and carbapenems (doripenem and imipenem**)**. Among them, gentamycin was used for the Columbia-based selective solid medium, and doxycycline was chosen to obtain our *Asaia*-free mosquito line.

### Natural *Asaia* spp. infection in wild flowering plants

Flowers were collected from 11 endemic and widely distributed plant species (Table 2) across the study area, including those mainly encountered inside and around the villages of Dielmo and Simong. Among the collected flowers, only those of *Datura metel* 25% (2/8) and *I. pes-caprae* 25% (28/114) were found to be contaminated with *Asaia* spp. *I. pes-caprae* were collected at three different locations (Table 2); this was the most targeted plant due to its wide distribution in the study area and its proximity to mosquito breeding sites.

**Table 2:** Plant collection and *Asaia* spp. carriage by plant species.

Plants species	Collection sites	Positive	Negative	Total	Proportion
*Ludwigia leptocarpa*	Dielmo	0	70	70	0
*Commelina benghalensis*	Dielmo	0	7	7	0
*Ageratum conyzoides*	Dielmo	0	15	15	0
*Kohautia grandiflora*	Dielmo	0	10	10	0
*Oldenlandia lancifolia*	Dielmo	0	33	33	0
*Anacardium occidentale*	Dielmo	0	30	30	0
*Datura metel*	**Dielmo**	**2**	**6**	**8**	**25%**
*Nesaea radicans*	Dielmo	0	0	34	0
*Polygonum salicifolium*	Dielmo	0	0	33	0
*Urena lobata*	Dielmo	0	0	10	0
*Ipomea pes-caprae*	**Nema edges**	**12**	**68**	**80**	**15%**
*Ipomea pes-caprae*	**Simong edges**	**4**	**10**	**14**	**28.6%**
*Ipomea pes-caprae*	**Dielmo**	**12**	**8**	**20**	**60%**

**Figure 1:**
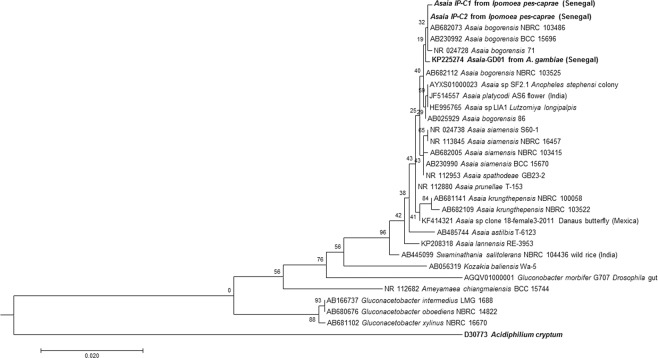
Phylogeny of the *A. aff. bogorensis* strains isolated from *I. pes-caprae*. Figure 1 displays the molecular phylogenetic evolutionary reconstruction of the Senegalese strains of *Asaia* isolated from *I. pes-caprae* (*Asaia IP*-*C*1 and *Asaia IP-C*2) and those from *An. gambiae s.l*. from Dielmo (*Asaia* GD01) (in bold). The branch lengths are proportional to the genetic distance between the strains, considering the scale placed below the tree. The numbers in front of the nodes are the bootstrap values. The names of the reference strains retrieved from Genbank start with their accession number followed by the species name and the strain reference when available.

The colonies cultivated from all the infected specimens of *I. pes-caprae* that were morphologically similar to those of *A. aff. bogorensis* GD01, previously isolated by our team from *An. gambiae s.l*. collected from Dielmo (Senegal), were confirmed by qPCR^2^. The qPCR molecular screening showed that two colonies out of a total of 20 morphologically similar colonies isolated on *Asaia*-selective antibiotic-containing solid medium tested positive for *A. aff. bogorensis*. The subsequent phylogenetic analysis based on the sequences of almost the entire 16 S rRNA gene confirmed that the isolated strains were *A. bogorensis*, with slight differences from *A. aff. bogorensis* GD01, which was previously isolated from *An. gambiae s.l*. in the same village (Fig. 1), forming a clade within the *A. bogorensis* cluster.

### Natural prevalence of *Asaia* spp. in a laboratory mosquito strain

A subsample of 158 *An. coluzzii* (130 females and 28 males) of the laboratory strain were randomly selected and screened to determine the prevalence of natural infection by *A. aff. bogorensis* in our experimental study population. The results showed a global carriage rate of *A. aff. bogorensis* up to 61% (96/158), which was significantly higher among the female group (74%,96/130) than among the male group (46.4%,13/28)(χ^2^ = 6.864, df = 1, *p* = 0.008). The naturally infected *An. coluzzii* laboratory strain was used as the *Asaia*-positive line in the following experiments.

### Experimental infection model

qPCR screening of newly opened *Pseuderanthemum reticulatum* flowers used in the experimental infection revealed the absence of natural *Asaia* infection. The plants were then isolated and protected from contact with arthropods. A batch of 100 *An. coluzzii* individuals, randomly selected from a naturally *Asaia*-infected laboratory colony, were exposed to *P. reticulatum* to confirm the effectiveness of transmission. During the entire experimental period, the only source of food for mosquitoes was the plant nectar, to which they were exposed for 3 consecutive days. Screening for *Asaia* infection on 36 randomly selected open flowers revealed the presence of *Asaia* spp. in 31/36 (86.1%) of the flowers. This shows that mosquito nectar feeding could be a significant route of bacterial contamination of plant flowers.

PCR screening of a randomly selected subsample of 56 anopheles (30 females and 26 males) out of the 200 *An. coluzzii* treated with antibiotics for 3 days to establish an *Asaia*-free lineage revealed no infection, thus confirming the *Asaia*-free status of the batch. The remaining females were reared to obtain the first generation (F1) of the lineage used for the following experiments. Samples of the *Asaia*-free lineage, selected randomly and in a timely manner, were regularly tested to verify whether reinfection with *Asaia* occurred. The lineage remained pure and uncontaminated throughout the experiments.

**Figure 2:**
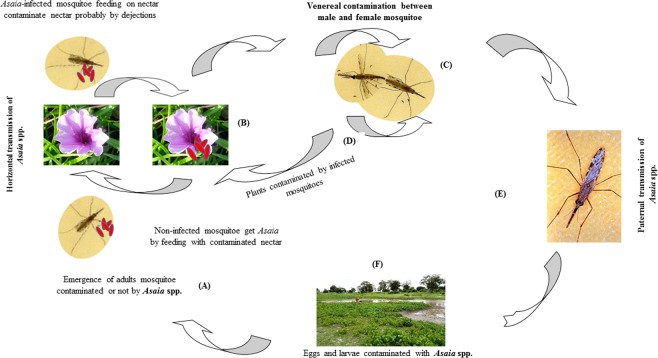
Putative *Asaia* spp. natural transmission cycle involving endemic plants in the field. (**A**) Emerging adult mosquitoes (infected or not) feed on plants to obtain the nectar needed for their survival and ensure reproduction. During the diet or oral (potentially co-feeding) transmission phase (**B**), mosquitoes are contaminated or contaminate the plants they feed on. Then, during the copulation or sexual transmission phase (**C**), male or female mosquitoes can contaminate their partners. The fertilized females will search for a blood meal to ensure the maturation of their eggs (**D**), and the males will continue to feed on nectar from plants, such as *I. pes-caprae*, to satisfy their sugar needs and maintain the oral transmission cycle (**E**). After a blood meal, the females will lay eggs (**F**), some of which will be contaminated by *Asaia* spp. as well as larvae (known as the transstadial transmission phase).The infected emerging adults will initiate and maintain the transmission cycle.

The subsequent exposure of two replicates of 265 *An. coluzzii* specimens belonging to the *Asaia*-free laboratory colony to *P. reticulatum* plants previously infected by the *Asaia*-positive lineage revealed a contamination rate of 27% (71/265) (Table 3). The infection rate was significantly lower in the female group (16.0%, 24/153) than in the male group (42.0%, 47/112) (χ^2^ = 21.45, df = 1, *p* < 0.05). These results have enabled us to propose a transmission cycle of *Asaia* spp. among mosquitoes in the natural environment (Fig. 2).

**Table 3:** Natural prevalence of *A. aff. bogorensis* GD01 in the laboratory colony and experimental model in 2017 and 2018.

Year	Natural prevalence of *A. aff. bogorensis* GD01 in the laboratory *A. coluzzii* colony			Experimental infection model		
	Males	Females	Total	Males	Females	Total
2017	54% (15/28)	76.15% (99/130)	72.15% (114/158)	34.43% (21/61)	14% (9/64)	24% (30/125)
2018	46.4% (13/28)	74% (96/130)	61% (96/158)	42.0% (47/112)	16% (24/153)	27% (71/265)

## Discussion

Arthropods maintain a complex relationship with the elements present in their natural environment. The impact of these elements on the transmission of vector-borne diseases has long been under-researched. However, nectar-producing plants play a very important role in the transmission of pathogens hosted by nectarivorous arthropods. Nectar is one of the main sources of food for insects and helps to increase their survival in the natural environment. As in larval breeding sites, where some bacteria belonging to the microbial flora can be ingested by mosquito larvae^13,24^, bacteria present in plant nectar can also pass into adult mosquitoes during nectar feeding. Bacterial species, such as those belonging to the *Asaia* genus, represent great potential for use in biological malaria control, especially in the current context of the emerging resistance of *P. falciparum* to artemisinin^25–27^ and of the main vectorial species of malaria to insecticides^28–31^. *Asaia* bacteria belong to the *Acetobacteraceae* family and are often isolated from fermented foods or plants^32,33^. They have also been described as symbionts of insects that often feed on natural sources of sugar^34,35^.

*Asaia* spp. could be modified by trangenesis to obtain a strong antagonistic effect against *P. falciparum*^9,36^ and may be able to protect mosquitoes against plasmodial infection. This antagonistic effect was first highlighted by Favia *et al*.^9,37^. Furthermore, the *Asaia* bacterium has been found to be stably associated with the microbiota of larvae and adults of *An. stephensi*, *An. maculipennis* and *An. gambiae*^37,38^. This bacterium has also been found to be stably associated with the microbiota of the laboratory strain of *An. coluzzii*, and naturally infect *An. funestus*^2^. Online searches in the metagenomic 16 S databases have also shown the presence of *Asaia* in different nectarivorous insects, such as butterflies and bees, in which the most likely propagation pathways identified were the venereal route, paternal transmission^39,40^ and food ingestion^41^.

*Asaia*, a genus of acetic acid bacteria was first discovered and isolated from plants, namely, *Bauhinia purpurea* and *Plumbago auriculata* flowers^42^. Currently, only 8 species of *Asaia* have been described: *Asaia astilbis*^43^, *Asaia bogorensis*^42^, *Asaia krungthepensis*^18^, *Asaia lannensis*^44^, *Asaia platycodi*^43^, *Asaia prunellae*^43^, *Asaia siamensis*^16^, and *Asaia spathodeae*^45^. To our knowledge, no studies have been able to highlight the role of plants in the contamination of arthropods by *Asaia* spp. In the study of tularemia, scientists have shown through an experimental model that nectar may play a key role in the spread of the disease. Indeed, *Francisella tularensis* has the ability to survive in nectar, thus facilitating its ingestion by vectors, which are then infected through the nectar feeding route, thus ensuring the transmission of the bacteria^14^. *Asaia* strains were found in the nectar of some plants in this study, suggesting that infected plants could be the missing link to the maintenance of mosquito infection in nature. Our hypothesis is reinforced by previous reports on the ability of bacteria to survive in plant nectar^14^. Using both molecular and microbiological methods, we demonstrated natural infection of flowers of *I. pes-caprae* and *D. metel* by *Asaia* bacteria. The existence of viable *Asaia* strains in *I. pes-caprae* flowers was demonstrated using bacterial isolation techniques with homemade selective medium. Moreover, we established an *in vivo* transmission model demonstrating the key role played by plants in the natural transmission of *Asaia* to *Anopheles* via contaminated nectar.

Together, the high prevalence of *Asaia* spp. in plants in the direct environment of mosquitoes (*I. pes-caprae* growing close to mosquito breeding sites and *D. metel* found in the vicinity of human dwellings, which also serving as adult mosquito resting sites) and the transmission of *Asaia* from infected to noninfected *An. coluzzii* mosquitoes via flower nectar provide enough evidence to support our hypothesis. Based on this evidence, we suggest the transmission cycle for *Asaia* spp. in the natural environment (Fig. 2). In addition to mosquitoes, the carriage of *Asaia* spp. is widespread in nectarivorous insects in nature. Indeed, *Asaia* spp. have already been isolated or identified in *Anopheles* mosquitoes^46^, monarch butterflies (*Danaus plexippus*, Genbank accession number KF414321), honey bees^47^, *Ae. aegypti*, *Apis mellifera*, *Drosophila melanogaster*, *Saccharicoccus sacchari*^39^ and many other insects^35,48^.

In the context of the spread of insecticide resistance and the increased need for innovative approaches to control vectors, the development of genetically modified mosquitoes (GMMs) that are resistant to pests has emerged as the most promising alternative to insecticide interventions^49^. However, the GMM approach has one main limitation: it modifies the fitness and competitiveness of mosquitoes in relation to natural mosquito populations^9^. Given the recent evidence of the stable natural infection of malaria vectors by several bacteria that may impede *Plasmodium* development, *Asaia* spp. have become one of the most promising potential paratransgenic weapons against malaria^9^. *Asaia* spp. are easily cultivable and can be genetically manipulated to deliver anti-pathogen effects on molecules that are then reinserted into their insect host^36^. A prominent model of the paratransgenetic malaria control strategy based on *Asaia* bacteria has been recently proposed^3^. *Asaia* bacteria have been genetically modified to release scorpine into the intestinal lumen of *An. stephensi*, leading to the elimination of *P. berghei*.

We have noticed that in laboratory colonies of *An. coluzzii*, male mosquitoes are more likely than females to be infected by *Asaia*. We therefore speculate that the females (probably fertilized) were in search of a blood meal for maturing their eggs and, therefore, consume less *Asaia*-infected nectar. However, this hypothesis was not tested in the present study.

*In fine*, this study is an important steppingstone toward the successful use of any *Asaia*-based vector control approach. Given the strong antimalarial capacities of these bacteria, the involvement of plants as a key link between nectarivorous insects, including several hematophagous arthropods that transmit vector-borne diseases, in the natural transmission cycle of *Asaia* bacteria offers an unprecedented opportunity for the direct or indirect use of *Asaia* in an alternative biological and/or transgenic approach to malaria control.

## Material and Methods

### Description of the study area and plant collection

**Figure 3:**
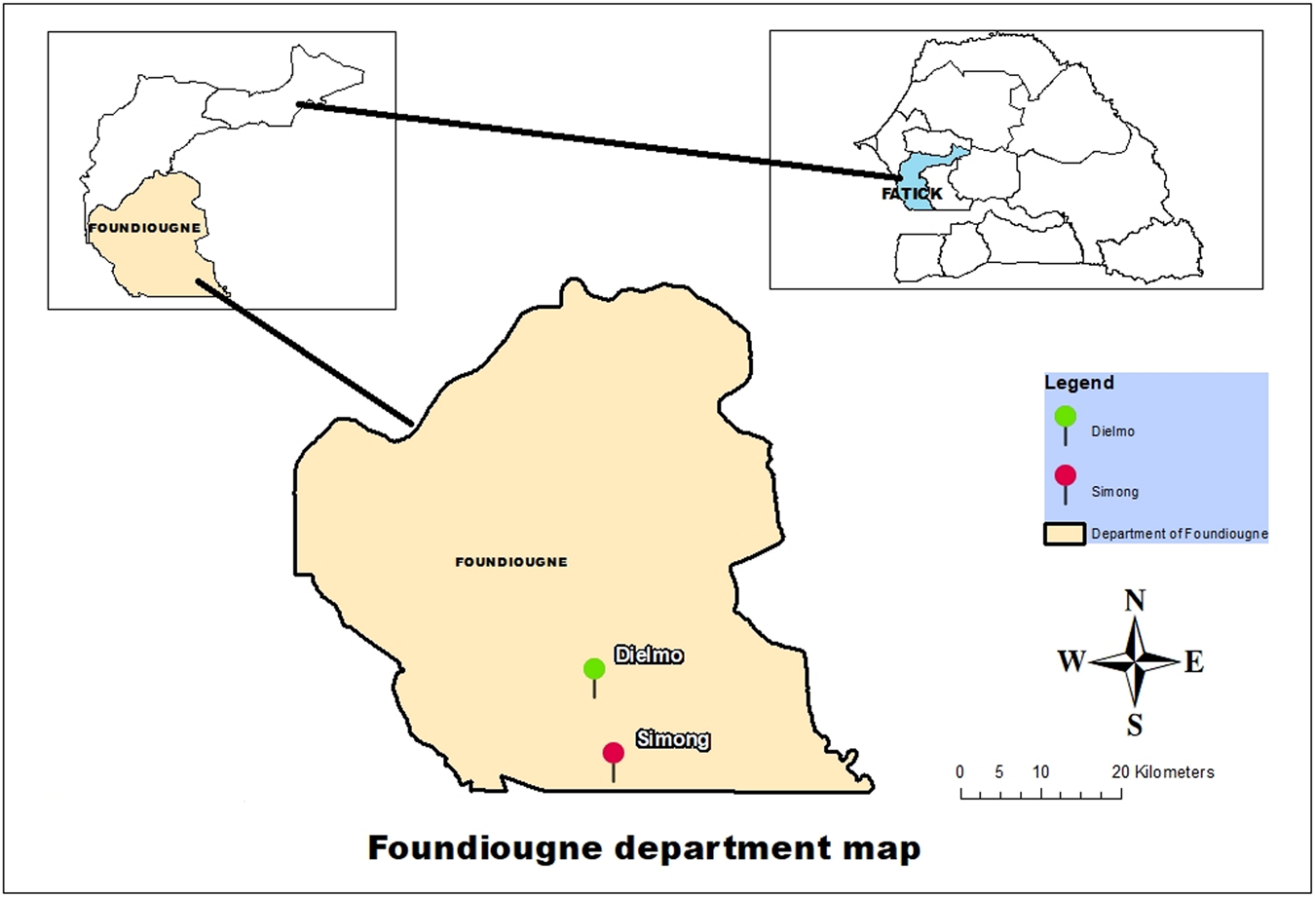
Map of the study sites. The positions of the two collection sites are indicated by red (Simong) and green (Dielmo) dots. Both villages are close to the Saloum delta, one of the most irrigated areas in Senegal.

Entire flowers were manually collected from endemic plants growing on the banks of the Nema River, next to Dielmo (13°43′23.43′′N, 16°24′46.27′′W) and Simong (13°37′55.63′′N, 16°23′08.24′′W) (Fig. 3). Dielmo is located in the Fatick region of Senegal, in an area of Sudanese savannah approximately 174 miles from Dakar and 9.32 miles north of the Gambian Republic. Since 1990, the area near Dielmo has hosted one of the oldest cohort studies investigating the epidemiological relationships between the malaria parasite and its vectors and human hosts^50^. Previous studies have already laid out the geographical and epidemiological characteristics as well as the dynamics of malaria in Dielmo^50,51^.

Members of the *An. gambiae s.l*. and *An. funestus* group are the main vectors of malaria in this area^52,53^.

The flowers were collected from 27 to 30 October 2016. They were cut at the stalk level, placed in bags (one bag per plant), stored at 4 °C in the Dielmo laboratory and transported to Dakar at the same temperature. In Dakar, the samples were immediately frozen at −80 °C until they were processed to assess their molecular biology and for bacterial cultures.

### Molecular analyses

#### DNA extraction

The petals and sepals of each flower were removed, and then the peduncle was cut off. The remaining flower parts, including nectar and pistil, were immersed in an Eppendorf tube containing 600 μl of 1X phosphate buffered saline (PBS) and then crushed. After centrifugation, 200 μl of the supernatant was collected for DNA extraction, which was performed according to the 2% CTAB method^54^ supplemented with β-mercaptoethanol at a concentration of 0.2%. The mixture was left to digest overnight. The same DNA extraction procedure was performed for the mosquitoes. Before DNA extraction, individual specimens were sacrificed at −80 °C, washed with 70% ethanol to remove superficial environmental bacteria and then crushed. Then, genomic DNA was immediately extracted from the whole body of each individual specimen as previously described^54^.

#### *Asaia* bogorensis molecular detection

Taqman® qPCR methods with custom designed primers and probe sets were used to screen samples for the presence of *Asaia* spp. by targeting the *rpoB* gene (Fwd: 5′-GACGCCAAGGACCTGATCTA-3′; Rev: 5′-ATAGGCCAGGATTTCGTCCT-3′; Probe: 6-FAM-GGTCACGACCCTGCTCTATG-TAMRA)^2^. The targeted region was amplified in a total reaction volume of 20 μl, containing 10 μl of ROCHE® master mix (Roche Diagnostics, Indianapolis, IN, USA), 0.5 μl of each primer, 0.5 μl of the probe, 3 μl of distilled water, 0.5 µl of UDG, and 5.0 μl of test DNA. The conditions of the amplification with the CFX96 Touch detection system (Bio-Rad, Marnes-la-Coquette, France) were as follows: 2 minutes at 50 °C for UDG action followed by an initial denaturation of 5 minutes at 95 °C, then 39 cycles of 5 seconds at 95 °C and 30 seconds at 60 °C. A sample was considered positive when the Ct value was less than 35 cycles. The Anopheles negative control was prepared from a crush of certified *Asaia*-free *An. coluzzii*, and the positive control was prepared from *An. coluzzii* to which we added a suspension of *A. aff. bogorensis* GD01.

#### 16 S rRNA gene sequencing and phylogenetic tree construction

The molecular phylogenetic evolutionary history was inferred from a nearly complete 16 S rDNA sequence (1249 bp), which was sequenced using universal 16 S rDNA primers fD1 and rp2^55^, as described by Drancourt *et al*.^56^.

Initially, we used a Peltier PTC-200 model thermal cycler (MJ Research Inc., Watertown, MA, USA) to perform the PCR amplifications. The reactions were carried out using HotStar Taq polymerase (Qiagen, Hilden, Germany) following the manufacturer’s instructions. For each assay, we included negative and positive controls for the validation of the run. The success of amplification was confirmed by electrophoresis on a 1.5% agarose gel.

The purification of the PCR products was performed using NucleoFast 96 PCR plates (Macherey-Nagel EURL, Hoerdt, France) according to the manufacturer’s instructions. The products of the amplification were then sequenced using a Big Dye Terminator Cycle Sequencing Kit (Perkin Elmer Applied Biosystems, Foster City, CA) with an ABI automated sequencer (Applied Biosystems).

The amplified sequences were assembled and corrected using ChromasPro software (ChromasPro 1.7, Technelysium Pty Ltd., Tewantin, Australia) and then compared with reference sequences available in Genbank using the BLASTN server (http://blast.ncbi.nlm.nih.gov/Blast.cgi) to identify closely related species and/or strains.

The taxonomic relationships of the new strains were inferred against the existing isolates. The reference sequences retrieved from the Genbank database, together with the Senegalese strains, were aligned using the ClustalW multisequence alignment program^57^ in BioEdit software^58^. A maximum likelihood phylogenetic tree was reconstructed using TOPALi v2.5 based on the Hasegawa-Kishino-Yano (HKY85) substitution model^59^, which includes the proportion of invariable sites and the gamma distribution. The robustness of the individual branches was estimated by bootstrapping with 100 replicates^60^.

### Establishment of an *Asaia*-free *An. coluzzii* strain

#### Sensitivity of A. aff. bogorensis GD01 to antibiotics

The sensitivity of the *A. aff. Bogorensis* GD01 strain, previously isolated from *An. gambiae* from Dielmo village^2^, to antibiotics was determined. A pure suspension of *A. aff. bogorensis* GD01 at a concentration equal to the 0.5 McFarland standard was inoculated onto Mueller-Hinton agar medium^61,62^. Antibiotic (amoxicillin, amoxicillin/clavulanic acid, piperacillin/tazobactam, doripenem, imipenem, aztreonam, ceftriaxone, ceftazidime, cefpirome, gentamicin, doxycycline, erythromycin, trimethoprim/sulfamethoxazole, ciprofloxacin, linezolid, metronidazole and rifampicin) discs were deposited onto dried agar plates and the plates were then incubated at 28 °C under 5% CO_2_ atmosphere. Finally, the inhibition diameters were measured and compared with the reading charts according to the manufacturer’s instructions (i2a - Siège Social, 401 Avenue du Walhalla, CS83406, 34060 Montpellier Cedex 2,France) and the recommendations of the Antibiogram Committee of the French Society of Microbiology^63^.

#### Isolation of strains of Asaia spp. from wild plants

Suspensions of the qPCR-positive *I. pes-caprae* were seeded on Columbia agar supplemented with 5% fresh sheep blood and 4 mg/l gentamicin (batch No. F140512, manufactured by Xin K. Pharm Co. Ltd). The seeded medium was then incubated at 28 °C under an atmosphere of 5% CO_2_. The colonies morphologically resembling *Asaia* were seeded on another Columbia agar plate supplemented with 5% fresh sheep blood but without antibiotics and incubated under the same conditions. Approximately 50 purified colonies were then collected and suspended in 200 μl of sterile 1X PBS and used for bacterial DNA extraction using the 2% CTAB method supplemented with 25 μl of proteinase K (*Tritirachium album*, 25 mg, ref. EUC0090-A, EUROMEDEX, 24, rue des Tuileries BP684 67460 Souffelweyersheim, France). Finally, the extracted DNA was screened for the presence of *Asaia* using *A. bogorensis*-specific qPCR.

#### Treatment of adult mosquitoes with antibiotics

Adult *An. coluzzii* colonies maintained at a temperature of 26 ± 2 °C and a relative humidity of 80 ± 10% in our laboratory, as described previously^50–53^, were fed a mixture of sterile water containing doxycycline (batch No. B113304 manufactured by SERB Laboratories) at a concentration of 8 mg/l and 10% sucrose solution for 4 days. qPCR was performed on the treated mosquitoes to confirm their *Asaia*-free status.

#### Insectary rearing of the *Asaia-*free mosquito strain

The *Asaia*-free mosquito line, fed sugar solution prepared with autoclaved water, was maintained at a temperature of 26 ± 2 °C and a relative humidity of 80 ± 10%. When feeding the mosquitoes, a volume of 50 ml of autoclaved water was filtered with a 0.2 µm filter and then mixed with sterile sucrose solution at a concentration of 10%. This mixture was offered daily to mosquitoes and changed daily to prevent mosquito reinfection. The physical conditions of breeding were the same as those of the other mosquitoes in the insectarium. The control of the absence of *Asaia* in this mosquito line was assessed weekly and before each experiment using our *Asaia*-specific custom-designed qPCR system.

#### Design of the experimental infection through transmission of *A.aff. bogorensis*

The two *An. coluzzii* colonies (*Asaia*-infected and *Asaia*-free), maintained separately in the insectarium of the VITROME laboratory of the Institut de Recherche pour le Développement (Dakar, Senegal), were used for the experimental infection of *A. aff. bogorensis* transmission. The common ornamental plant *P. reticulatum* (*Acanthaceae*) was selected as the source of nectar during the experimental infection study after the confirmation of its natural *Asaia*-free status and due to its easy procurement from urban horticulturists in Dakar, where our laboratory is based. The plants were grown in pots within a closed room with no access to flying arthropods to prevent the contamination of newly opened flowers with *Asaia*. Randomly selected flowers from each plant were screened for the presence of *Asaia* spp. and only plants that were negative were used for the subsequent experimental infection.

Overall, 310 mosquitoes of the naturally *Asaia-*infected laboratory colonies (123 males and 187 females) were brought into contact with an uninfected *P. reticulatum* bearing newly opened flowers. The plant in its pot was introduced into a closed 100 × 50 × 50 cm mosquito cages for 3 days. During the experimental infection, the average temperature of the standard thermo-hygronomic device was 26 ± 2 °C, and the relative humidity was 80 ± 10%. At the end of the third day, the mosquitoes were sacrificed at −80 °C and then screened with randomly selected flowers using our qPCR method to confirm that the mosquitoes and the flowers of the exposed plants were infected with *Asaia*. The following day, an unfed batch of mosquitoes from the *Asaia*-free lines was allowed to feed for 3 days on the *Asaia*-infected plants under the same conditions described above. After exposure, the mosquitoes were sacrificed, stored individually in Eppendorf tubes for DNA extraction and then examined for *Asaia* infection. This experiment was repeated twice with the same number of anopheles for both lineages and the same plant species under identical physical conditions.

### Statistical analyses

This study was carried out over two consecutive years. Two groups of mosquioes were used each time. This first group was the *An. coluzzii* strain naturally infected by *A. aff. bogorensis* and the second was as *An. coluzzii Asaia*-free group obtained after antibiotic treatment and then exposed to infected *P. reticulatum*. Within each group, we analyzed the differences in infection rates between the two sexes. Statistical analyses were performed with Epi Info software version 7.0.8.8.0 (Centers for Disease Control and Prevention, Atlanta, GA, USA). The differences were analyzed using the Yates corrected χ2 test with one degree of freedom and a 95% confidence interval. The difference was considered significant when the bilateral *p* value < 0.05
